# Can alterations in cathepsin levels restrain the development of skin cancer?: A bidirectional multivariate Mendelian-randomization study

**DOI:** 10.1097/MD.0000000000039628

**Published:** 2024-09-20

**Authors:** Fan Bu, Kai Yu, Changtao Ye, Guixia Huang, Tianye Yang, Kang Chen, Ji Lu, Li Rong

**Affiliations:** a Department of Plastic and Aesthetic Surgery, The First Hospital of Jilin University Changchun, Jilin, China; b Department of Urology, The First Hospital of Jilin University Changchun, Jilin, China.

**Keywords:** basal cell carcinoma, cathepsin, cutaneous squamous cell carcinoma, melanoma, Mendelian randomization

## Abstract

Malignant skin tumors mainly include basal cell carcinoma, squamous cell carcinoma, and malignant melanoma. There is currently observational research suggesting that changes in cathepsin (CTS) may be a factor in the development of malignant skin tumors, but no studies have yet demonstrated a causal relationship between tissue protease changes and the occurrence of malignant skin tumors. Current studies have shown that cathepsin is involved in tumor cell invasion and metastasis by regulating growth factors and cellular immune function in tumor microenvironment, decomposing extracellular matrix and basement membrane, and promoting angiogenesis. In this study, we conducted a bidirectional Mendelian-randomization study using publicly available genome-wide association study (GWAS; GWAS Catalog) data. This study applies a bidirectional multivariate Mendelian randomization (MR) approach to investigate the causal relationship between cathepsin, basal cell carcinoma, squamous cell carcinoma, and malignant melanoma. In cases where multiple cathepsins are implicated as etiological factors in certain diseases, a multivariable analysis is conducted to assess the direct and indirect causal effects of the exposure factors. In this study, we present a comprehensive MR analysis to investigate the relationship between 9 cathepsin and basal cell carcinoma, squamous cell carcinoma, and malignant melanoma. Based on our MR analysis using the largest GWAS Catalog dataset available, we are able to draw relatively reliable conclusions. In the MR study, we found that tissue protease L2 can promote skin cancer, Cathepsin O, and Cathepsin F are associated with an increased risk of basal cell carcinoma. Cathepsin H can inhibit basal cell carcinoma and malignant melanoma. In the reverse MR study, it was found that squamous cell carcinoma may cause an increase in Cathepsin O expression. In the multivariate analysis, it was found that Cathepsin H is a direct factor in reducing the occurrence of skin cancer and melanoma, with no apparent causal relationship to non-melanoma skin cancer. Cathepsin has a dual impact on skin cancer cells, and the expression of different cathepsins at the edge of skin tumors may indicate different developmental tendencies of skin cancer. Cathepsin may serve as effective biomarkers for predicting tumors.

## 1. Introduction

Skin cancer can be divided into melanoma and non-melanoma skin cancer. Melanoma primarily includes malignant melanoma, while non-melanoma skin cancer mainly includes basal cell carcinoma (BCC) and cutaneous squamous cell carcinoma (CSCC). According to the American Cancer Society’s 2023 statistics.^[1]^ In the United States, melanoma ranks as the fifth most common cancer, with an estimated 97,610 new cases expected in 2023, of which approximately 60% are in males. In terms of melanoma mortality, the rate in males is about 2.1 times that of females. In China’s 2022 melanoma incidence statistics, an estimated 8114 new cases are expected, with no significant gender differences in incidence and mortality rates.^[2]^ Malignant tumors primarily occur on the surface of the skin, and their invasion and metastasis depend on the hydrolysis of the extracellular matrix surrounding the tumor. The activity of intracellular cathepsin in skin tumor cells is closely related to this process.^[3]^

Cathepsins are a group of lysosomal proteases.^[4]^ Cathepsins, including S, F, G, H, B, O, E, Z, and L2, among others, play crucial roles in promoting the invasion and metastasis of tumor cells through various mechanisms. These mechanisms encompass degradation of the extracellular matrix and basement membrane, modulation of the tumor microenvironment, impact on immune cell function, and facilitation of angiogenesis.^[5]^ Each cathepsin exerts a distinct influence on the development of skin malignancies based on its unique biological properties. For instance, Cathepsin L2,^[6]^ B,^[7]^ F,^[8]^ and S^[9]^ contribute to extracellular matrix remodeling and the modulation of interactions between tumor cells and their microenvironment, thereby influencing skin malignancy progression. Additionally, Cathepsin G^[10]^ and Cathepsin Z^[11]^ regulate immune cell function, indirectly affecting tumor growth and dissemination in skin malignancies. Cathepsin H^[12]^ influences skin malignancy development by modulating signaling and activity within tumor cells. The precise roles of Cathepsin O and Cathepsin E in skin malignancies warrant further investigation. Cathepsin has been confirmed to be involved in the development of various cancers, including breast cancer,^[6]^ lung cancer,^[7]^ and colon cancer.^[8]^ Cathepsin is known to participate in various cellular functions within the skin, including intracellular protein processing, epidermal homeostasis, and hair development.^[9]^ However, it is still unclear whether cathepsin can cause malignant tumors in the skin. Due to differences in the composition and genetic sequence of proteases,^[10]^ their roles in malignant tumors in the skin also vary. The causal relationship between different types of cathepsin and the risk of various histological skin cancers has not been fully studied. Therefore, further research is necessary to investigate the causal relationship between different types of cathepsin and the risk of various skin cancers.

This study used Mendelian randomization (MR) to explore the causal relationship between cathepsin and skin cancer.^[11]^ Currently, research on the association between cathepsin and skin cancer is relatively limited, and large-scale, multi-sample studies have not been conducted. Existing research may be influenced by unknown factors.^[12]^ MR uses genetically related single-nucleotide polymorphisms (SNPs) as instrumental variables for cathepsin acting on related diseases, to evaluate the causal relationship between cathepsin as an exposure factor and outcomes. MR has the advantage of large sample size and being unaffected by confounding factors, and has been widely used in exploring the etiology of diseases.^[13]^ In this study, comprehensive statistical data from the genome-wide association study (GWAS) Catalog database were used to conduct 2-sample multivariate MR analysis. For diseases that have multiple positive exposure factors simultaneously, multivariate research was conducted to identify the potential direct exposure factors of cathepsin for various types of skin cancer.

## 2. Methods

### 2.1. Instrumental variables acquisition

The cathepsin data involved in this study are derived from the INTERVAL study by Jialin Li.^[14]^ The cathepsin mainly include 9 types of cathepsins (S, F, G, H, B, O, E, Z, L2), involving a total of 3301 European individuals. The data mentioned in the article can be obtained from https://www.ebi.ac.uk/gwas/downloads/summary-statistics and http://ftp.ebi.ac.uk/pub/databases/gwas/summary_statistics/. Since we solely used previously published data from public databases, no additional ethical approval was required for this study. Selection of cathepsin-related instrumental variable (IV)s for MR analyses followed specific criteria: an r2 measure of LD among instruments <0.001 within a 10,000 kb window; *P* values below the genome-wide significant level identified as 5 × 10^−6^. To maintain the effectiveness of IVs, all SNPs selected by us have an *F* value greater than 10. The cathepsin-related data is shown in Table 1, and the SNPs selected based on the above criteria are presented in Supplementary Table 1, Supplemental Digital Content, http://links.lww.com/MD/N550.

**Table 1:** Related information on cathepsin in GWAS summary database.

GWAS ID	Year	Trait	Sample size	Number of SNPs
prot-a-718	2018	Cathepsin B	3301	10,534,735
prot-a-720	2018	Cathepsin E	3301	10,534,735
prot-a-721	2018	Cathepsin F	3301	10,534,735
prot-a-722	2018	Cathepsin F	3301	10,534,735
prot-a-723	2018	Cathepsin G	3301	10,534,735
prot-a-725	2018	Cathepsin H	3301	10,534,735
prot-a-726	2018	Cathepsin O	3301	10,534,735
prot-a-727	2018	Cathepsin S	3301	10,534,735
prot-a-728	2018	Cathepsin L2	3301	10,534,735
prot-a-729	2018	Cathepsin Z	3301	10,534,735

GWAS = genome-wide association study, SNP = single-nucleotide polymorphism.

### 2.2. Genetic association of SNPs with skin cancer risk

The data used in the study came from the improvement of the linear mixed model-based genome-wide association (GWA) by scholar Jiang et al.^[13]^ Summary statistics for skin cancer risk was obtained from the https://www.ebi.ac.uk/gwas/downloads/summary-statistics. Since we solely used previously published data from public databases, no additional ethical approval was required for this study. The obtained skin cancer data is shown in Table 2. Selection of cathepsin-related IVs for MR analyses followed specific criteria: an r2 measure of LD among instruments <0.001 within a 10,000 kb window; *P* values below the genome-wide significant level identified as 1 × 10^−5^. To maintain the effectiveness of IVs, all SNPs selected by us have an *F* value greater than 10. *F* value can be calculated through the following formula: *F* = *R*2(*N* − 2)/(1 − *R*2), where *N* means the sample size and *R*2 is the proportion of the variance of trait explained by the IVs. *R*2 = 2 × EAF × (1 − EAF) × *β*2, where EAF represents the effect allele frequency of IVs and *β* means the estimated effect of SNP. We can also obtain the *F* value in the following way [*F* = (*β*/se)^2^].

**Table 2:** Related information on skin cancer in GWAS catalog database.

First author	PubMed ID	Study accession	Trait	Cases	Controls	Sample size
Jiang et al^[13]^	34737426	GCST90041887	Skin carcinoma	1428	454,848	456,276
Jiang et al^[13]^	34737426	GCST90041829	Melanomas of skin	2824	453,524	456,348
Jiang et al^[13]^	34737426	GCST90041915	Non-melanoma skin carcinoma	665	455,611	456,276
Jiang et al^[13]^	34737426	GCST90041916	Basal cell carcinoma	4257	452,019	456,276
Jiang et al^[13]^	34737426	GCST90041917	Squamous cell carcinoma	557	455,719	456,276

GWAS = genome-wide association study.

### 2.3. Statistics in MR analysis

In the two-sample MR analysis, SNPs used as IVs should satisfy 3 key assumptions: IVs are strongly associated with exposure; IVs are independent of any confounding factors; IVs affect the outcome only through the exposure. When partial genetic variation causes a disease through factors other than the exposure, we consider this genetic variation to have horizontal pleiotropy, and do not believe that the exposure has a causal relationship with the disease.

In our study, we employed multiple different methods simultaneously to estimate the causal relationship between cathepsin and skin cancer ensure the accuracy of the research. The inverse variance weighted (IVW) method is the most widely used and valuable approach in MR, serving as the primary method to determine the validity of the exposure factor. The IVW method considers both fixed-effects and random-effects influences. As a meta-analysis approach, IVW obtained the total estimates of the effect of exposure on outcome by combining Wald estimates of causality for each IV. We also employed four supplementary methods to ensure the accuracy of the research results under different scenarios, including the MR-Egger regression, the weighted median, the simple mode, the weighted mode, and MR pleiotropy residual sum and outlier (MR-PRESSO) methods. Based on the assumption of Instrument Strength Independent of Direct Effect, the MR-Egger regression method performed a weighted linear regression. In our study, we considered an exposure factor to be valid if the *P* value of the IVW method was less than .05, and the direction of effect estimates from the remaining methods (all negative or all positive) was consistent. This scrupulous methodology aims to fortify the foundation of our understanding, ensuring the discernment of causality within the intricate tapestry of relationships between cathepsin and skin cancer.

Furthermore, the intercept term of MR-Egger regression method was considered as an indicator of directional pleiotropic effects, we consider the research results to be statistically significant when the *P* value is less than 0.05. Nevertheless, the MR-Egger regression method was relatively poor in examining the weighted median value of the ratio instrumental variable estimates. At last, the weighted mode method was used to assess the overall causal effect from a large number of genetic instruments. In addition to MR-Egger regression method, MR-PRESSO method was performed to test and process pleiotropy. We consider the research results to be statistically significant when the *P* value is less than .05. The number of distributions in MR-PRESSO analysis was set to default. Cochran’s statistic was calculated to quantify the heterogeneities detected by the IVW and MR-Egger regression methods, and a *P* < .05 was considered heterogeneous, and thus a random-effect model was applied for subsequent analyses. Otherwise, a fixed-effect model was used. Besides, the “leave-one-out “sensitivity analysis was applied for exploring whether there was a single SNP which created bias to influence the overall causal effect. The reverse MR uses skin malignant tumors as the exposure factor and cathepsin as the outcome factor. The multivariable MR analysis is much the same as the univariable MR analysis. The two-sample MR analysis was conducted using packages “Two Sample MR” and MRPRESSO open-source Statistical software R (version 4.2.3, R).

## 3. Results

### 3.1. Exploration of the causal relationship between cathepsin and skin malignant tumors

To assess the impact of different cathepsin on different types of skin malignant tumors, a univariate 2-sample MR analysis was first conducted on 9 cathepsins, including cathepsin B, E, F, G, H, L2, O, S, and Z, for different subtypes of skin malignant tumors. The univariate analysis results are presented in Table 3. Cathepsin L2 was found to have a promoting effect on skin cancer (odds ratio [OR] = 1.233, 95% confidence interval [CI]: 1.033–1.525, *P* < .05), while cathepsin F (OR = 1.048, 95% CI: 1.004–1.094, *P* < .05) and cathepsin O (OR = 1.053, 95% CI: 1.008–1.100, *P* < .05) were associated with an increased risk of BCC. Cathepsin H was found to inhibit BCC (OR = 0.9587, 95% CI: 0.9275–0.9909, *P* < .05), melanoma (OR = 0.9335, 95% CI: 0.8729–0.9983, *P* < .05), and CSCC (OR = 0.961, 95% CI: 0.946–0.976, *P* < .05). However, there was horizontal pleiotropy in the effect of Cathepsin H on squamous cell carcinoma (*P* < .05), and in this study, it is not considered that Cathepsin H can inhibit squamous cell carcinoma. No corresponding causal relationship between the remaining cathepsin and skin malignant tumors was observed.

**Table 3:** Causal association of cathepsins on skin cancer and its histological subtypes estimated by univariable Mendelian randomization analysis.

Outcome	Cathepsin	SNPs	Inverse variance weighted		Pleiotropy (Q_pval)		Heterogeneity	
			OR (95% CI)	*P* value	MR Egger	Inverse variance weighted	Egger_intercept	*P* value
Skin cancer	Cathepsin B	18	0.917 (0.801–1.050)	.211	0.217	0.240	−0.030	.487
	Cathepsin E	11	1.009 (0.829–1.228)	.930	0.548	0.508	0.046	.269
	Cathepsin F	11	0.945 (0.779–1.146)	.563	0.055	0.077	0.022	.707
	Cathepsin G	12	0.968 (0.803–1.168)	.736	0.560	0.642	−0.016	.789
	Cathepsin H	11	0.925 (0.845–1.012)	.090	0.530	0.602	0.012	.637
	Cathepsin O	11	0.932 (0.744–1.167)	.537	0.786	0.174	−0.126	.017
	Cathepsin S	24	1.011 (0.914–1.117)	.833	0.520	0.482	0.025	.216
	Cathepsin L2	10	1.255 (1.033–1.525)	.022	0.758	0.783	0.031	.474
	Cathepsin Z	11	0.962 (0.843–1.097)	.562	0.441	0.339	0.038	.165
Non-melanoma Skin carcinoma								
	Cathepsin B	18	1.154 (0.924–1.442)	.206	0.055	0.055	0.018	.538
	Cathepsin E	11	1.117 (0.837–1.49)	.452	0.436	0.436	0.095	.160
	Cathepsin F	11	0.946 (0.761–1.175)	.614	0.729	0.729	−0.073	.405
	Cathepsin G	12	1.224 (0.930–1.610)	.150	0.914	0.914	−0.065	.215
	Cathepsin H	11	0.910 (0.761–1.088)	.299	0.087	0.087	−0.010	.889
	Cathepsin O	11	1.190 (0.900–1.573)	.221	0.802	0.802	0.020	.761
	Cathepsin S	24	0.989 (0.854–1.145)	.881	0.526	0.526	−0.035	.555
	Cathepsin L2	10	1.238 (0.930–1.648)	.143	0.726	0.726	0.043	.413
	Cathepsin Z	11	1.045 (0.822–1.329)	.720	0.065	0.065	−0.063	.332
Basal cell carcinoma	Cathepsin B	21	0.986 (0.952–1.021)	.419	0.686	0.401	0.020	.032
	Cathepsin E	11	1.029 (0.960–1.103)	.418	0.548	0.118	0.038	.023
	Cathepsin F	19	1.048 (1.004–1.094)	.034	0.084	0.111	0.002	.889
	Cathepsin G	22	0.962 (0.924–1.002)	.061	0.973	0.967	−0.009	.317
	Cathepsin H	15	0.961 (0.934–0.987)	.004	0.310	0.246	0.010	.188
	Cathepsin O	19	1.053 (1.008–1.100)	.020	0.780	0.683	0.014	.142
	Cathepsin S	31	0.958 (0.924–0.993)	.021	0.109	0.002	0.022	.001
	Cathepsin L2	23	1.028 (0.976–1.084)	.293	0.011	0.014	0.006	.600
	Cathepsin Z	17	0.993 (0.955–1.031)	.700	0.561	0.626	−0.003	.765
Melanoma	Cathepsin B	18	0.968 (0.879–1.067)	.516	0.195	0.239	0.010	.754
	Cathepsin E	11	0.909 (0.770–1.073)	.258	0.181	0.169	−0.034	.331
	Cathepsin F	11	1.006 (0.905–1.118)	.911	0.446	0.541	0.000	.989
	Cathepsin G	12	0.981 (0.844–1.139)	.797	0.196	0.243	0.022	.649
	Cathepsin H	11	0.933 (0.873–0.998)	.044	0.299	0.369	−0.008	.701
	Cathepsin O	11	1.018 (0.889–1.166)	.793	0.697	0.778	0.003	.921
	Cathepsin S	24	1.042 (0.961–1.13)	.318	0.137	0.157	0.010	.556
	Cathepsin L2	10	1.013 (0.873–1.175)	.865	0.262	0.333	−0.012	.722
	Cathepsin Z	11	1.005 (0.920–1.098)	.911	0.550	0.609	0.011	.568
Cutaneous Squamous Cell carcinoma	Cathepsin B	10	0.961 (0.933–0.990)	.185	0.586	0.272	0.015	.066
	Cathepsin E	7	1.010 (0.950–1.074)	.417	0.890	0.890	0.006	.471
	Cathepsin F	8	0.977 (0.946–1.009)	.831	0.000	0.000	0.016	.392
	Cathepsin G	6	0.985 (0.918–1.057)	.942	0.862	0.422	−0.031	.129
	Cathepsin H	8	0.961 (0.946–0.976)	.004	0.704	0.048	0.013	.018
	Cathepsin O	6	1.057 (0.958–1.166)	.668	0.065	0.037	0.016	.310
	Cathepsin S	17	0.950 (0.929–0.971)	.083	0.000	0	0.005	.479
	Cathepsin L2	5	1.010 (0.928–1.099)	.585	0.434	0.326	0.013	.261
	Cathepsin Z	8	0.987 (0.957–1.017)	.859	0.002	0.002	0.007	.534

CI = confidence interval, MR = Mendelian randomization, OR = odds ratio, SNP = single-nucleotide polymorphism.

In the reverse causal study, melanoma was found to cause an increase in cathepsin E (OR = 1.051, 95% CI: 1.011–1.093, *P* < .05), while CSCC was found to cause an increase in cathepsin H (OR = 1.154, 95% CI: 1.045–1.273, *P* < .05) and cathepsin O (OR = 1.111, 95% CI: 1.006–1.226, *P* < .05). No heterogeneity and pleiotropy were observed in the positive results. There is no evidence to prove a causal relationship between the disease and other Cathepsin. The detailed content can be found in Supplementary Table 2, Supplemental Digital Content, http://links.lww.com/MD/N550.

**Figure 1.**
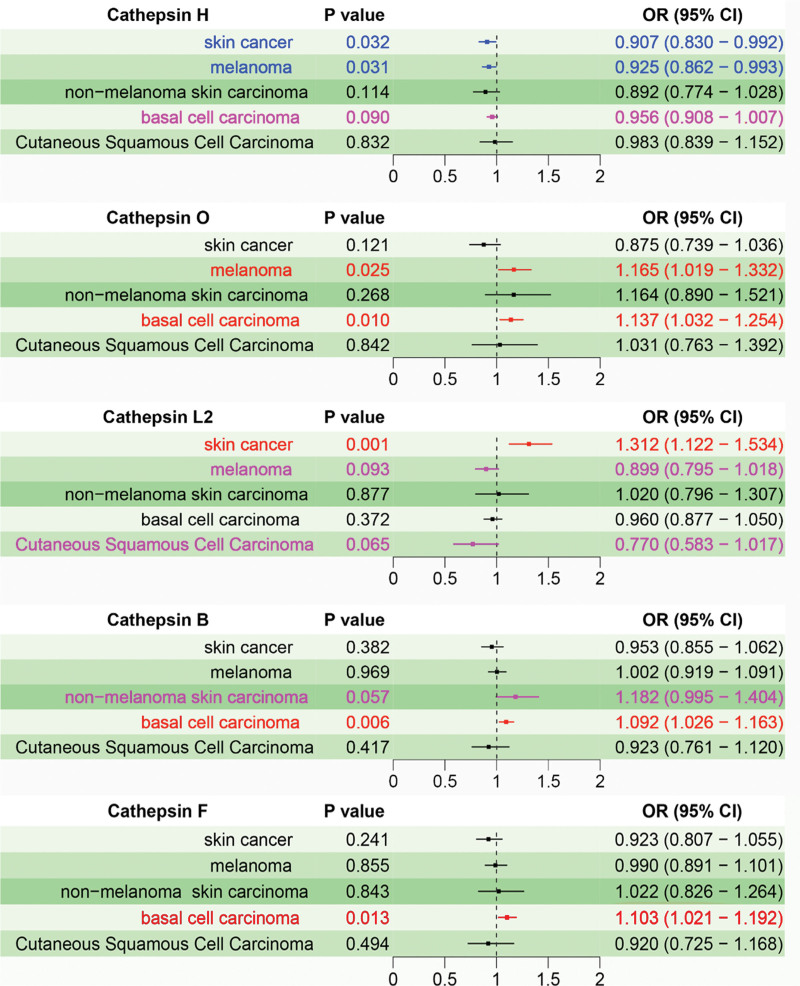
Multivariable analysis of cathepsin for skin cancer and its subclasses (red for promoting disease, blue for inhibiting disease, and pink for potential causation at *P* < .1). CI = confidence interval, OR = odds ratio.

In the multivariate study, Cathepsin H demonstrated independent inhibitory effects on skin cancer (OR = 0.907, 95% CI: 0.830–0.992, *P* < .05) and melanoma (OR = 0.925, 95% CI: 0.862–0.993, *P* < .05). Cathepsin O showed independent promoting effects on BCC (OR = 1.137, 95% CI: 1.032–1.254, *P* < .05) and melanoma (OR = 1.165, 95% CI: 1.019–1.332, *P* < .05). Cathepsin F exhibited a promoting effect on BCC (OR = 1.103, 95% CI: 1.021–1.192, *P* < .05). Cathepsin L2 had a significant promoting effect on skin cancer (OR = 1.312, 95%CI: 1.122-1.534, *P* < .05), and a promoting tendency for melanoma (OR = 0.899, 95% CI: 0.795–1.018, *P* = .09) and squamous cell carcinoma (OR = 0.770, 95% CI: 0.583–1.017, *P* = .06). Cathepsin B demonstrated a promoting effect on BCC (OR = 1.092, 95% CI: 1.026–1.163, *P* < .05) and a tendency for non-melanoma (OR = 1.182, 95% CI: 0.995–1.404, *P* = .06). Considering the results of the multivariate analysis and the bidirectional analysis, it is considered that Cathepsin H, L2, O, and B may have an impact on skin cancer and its subtypes. Detailed information can be found in Figure 1.

## 4. Discussion

Cathepsin are often distributed around the edges of tumors, as indicated in reference.[^16,17^] Additionally, the activity of cathepsin within tumor cells is positively correlated with pH levels, as mentioned in reference. [18] The invasion and metastasis of malignant tumors are closely related to the destruction of the extracellular matrix. Cathepsin have the ability to hydrolyze the extracellular matrix, but whether cathepsin can promote tumor proliferation, invasion, and metastasis is not yet clear. A comprehensive MR analysis was conducted to investigate the causal relationship between various cathepsins, specifically cathepsins S, F, G, H, B, O, E, Z, and L2, and skin malignancies, including BCC, squamous cell carcinoma, and malignant melanoma. Utilizing data from the GWAS catalog and employing bidirectional multivariate MR methods, the study elucidated several significant findings. Research indicates that particular cathepsin proteins, such as Cathepsin L2, promote skin cancer, while others, like Cathepsin H, exhibit inhibitory effects on BCC and malignant melanoma. Reverse causal analysis suggests that squamous cell carcinoma may upregulate the expression of cathepsin O, indicating a complex interplay between these proteases and skin cancer development. This study unveils the dual roles of cathepsin: some facilitate tumor growth and metastasis, while others act as potential biomarkers for early detection or therapeutic targets. By establishing a theoretical foundation for cathepsin-targeted diagnosis and therapy, the study aims to enhance patient care by deepening insights into how cathepsin influences the progression of skin cancer. The findings highlight cathepsin’s dual potential as both a promoter and an inhibitor of skin cancer, emphasizing the need for further research to discern these relationships and develop precise interventions.

The analysis of this study indicates that cathepsin H reduces the risk of patients developing skin cancer, melanoma, and BCC. However, high expression of cathepsin H is observed in squamous cell carcinoma. The results obtained using the IVW method are consistent with other supplementary methods. Cathepsin H is a type of lysosomal cysteine proteinase with unique amino endopeptidase activity.^[19]^ It has the function of degrading the extracellular matrix and regulating cellular signaling kinases.^[17,20]^ Its primary mechanism may involve promoting cancer invasion and metastasis through processes such as tumor–stroma interaction, extracellular matrix remodeling, and mediating degradation of engulfed matrix proteins within tumor cells.^[21]^

In reverse Mendelian research, it was found that skin squamous cell carcinoma can increase the expression of Cathepsin H. Cathepsin E and Cathepsin O are also increased in melanoma and skin squamous cell carcinoma. This indicates that cathepsin are involved in the immune response of the tumor microenvironment, and cathepsin inhibitors may become an important direction to inhibit the growth of malignant skin tumors. Cathepsin E is a type of lysosomal aspartic protease, primarily secreted by activated immune cells.^[22]^ Cathepsin E plays an important role in antigen presentation by major histocompatibility complex II cells in tumors, and it may participate in the tumor immune response within the tumor microenvironment, inhibiting the proliferation of tumor cells. Cathepsin O, on the other hand, is a type of cysteine protease that can be promoted by 20-hydroxyecdysone (20E) to enhance the immune response within the tumor. Its enzymatic activity can be inhibited by trans-epoxysuccinyl-L-leucylamido-(4-guanidino) butane (E-64).

This contributes to predictive diagnostics and early treatment for specific diseases. For diseases that may be caused by multiple factors, we have conducted multivariate analysis to determine whether cathepsin are directly causally related to the disease. Subsequently, we investigated the impact of the disease on cathepsin, which is helpful for auxiliary diagnosis and prognosis analysis. High expression of cathepsin in the disease may be associated with disease progression and metastasis.

Cathepsin B, a cysteine protease, plays a role in extracellular matrix breakdown and mucosal barrier disruption in disease.^[15]^ It is more expressed in less differentiated squamous cell carcinoma,^[16]^ with its involvement in invasion and its uncertain correlation with tumor stage. Cathepsin B contributes to the rat sarcoma pathway, affecting tumor proliferation and metastasis.^[17]^ Combining rat sarcoma and cathepsin inhibitors shows promise in treating head and neck squamous cell carcinoma. Cathepsin B also acts as a prognostic marker in melanoma, with variable expression in different tumors. Cathepsins B and D are mainly located in moderately differentiated head and neck CSCC tissue, while Cathepsin G is primarily located in mast cells.

The expression of Cathepsin L is significantly elevated in the cancer nests of BCC and SCC compared to normal tissue, with particularly notable increases in expression at the tumor margins. The detection of cathepsin can serve as an effective biomarker for tumor margins.

In addition to the aforementioned cathepsin, there are other cathepsin that play a role in the progression of skin malignancies. Cathepsin K is a cysteine protease with good collagen and elastin degradation capabilities. In normal skin, the expression of cathepsin K can be found in the stratum corneum, mature sebaceous gland cells, and the outer root sheath of hair follicles. Cathepsin K plays a key role in skin extracellular matrix degradation, crucial for tumor invasion and metastasis.^[18]^ Highly expressed in BCC,^[19]^ melanoma,^[20]^ and squamous cell carcinoma, it is particularly prominent in melanoma,^[21]^ the most metastatic. As a significant gene in the metastatic environment of CSCC, it promotes metastasis by inhibiting anti-tumor immune surveillance via the CD200-CD200R pathway.^[22]^

In our study, a 2-sample bidirectional multivariable MR analysis based on large-scale GWAS Catalog data was conducted to evaluate the causal relationship between cathepsin and malignant skin tumors and their subtypes. Compared to traditional observational studies, MR analysis can effectively reduce potential biases such as confounding factors and reverse causality, thereby enhancing the credibility of causal inference. The cathepsin and skin malignant tumor data used in this study are from the same region and population, significantly reducing the impact of characteristics from different regions.

While we employed various computational models and rigorous sensitivity analyses to improve the accuracy and reliability of the results, this study still has certain limitations. The study population included in our research is only from a single region, so further data analysis is needed to determine the generalizability of our conclusions to other regions. Additionally, due to genetic variation, we were unable to completely eliminate the influence of horizontal pleiotropy, so broader cathepsin data and studies involving more diverse populations are needed to minimize the impact of this factor on the results. Due to the abundance of cathepsin data, some SNP data are not yet available.

In summary, our MR analysis results suggest that the genetically predicted cathepsin H has an inhibitory effect on major subtypes of malignant skin tumors such as melanoma and CSCC, but further exploration is needed to understand its role in BCC. Melanoma and CSCC may play an important role in regulating the expression of Cathepsin E, H, and O at the tumor margins. This provides a theoretical basis for future development of cathepsin-targeted near-infrared fluorescence imaging to guide surgical margin delineation. To validate the accuracy of our results, future research will require the use of higher-quality GWAS data and more advanced methods.

## 5. Conclusion

This comprehensive MR study provides significant insights into the diverse functionalities of cathepsins in the context of skin cancer. The study reveals the dual role of cathepsins as both promoters and inhibitors of tumor development. Notably, cathepsin H demonstrates a potential protective effect against skin cancer, including melanoma and BCC, despite its elevated expression in squamous cell carcinoma. Conversely, cathepsins such as Cathepsin B and K are implicated in promoting tumor invasion and metastasis, emphasizing their critical role in the tumor microenvironment. The findings of this study suggest the exploration of cathepsins as potential biomarkers for predicting tumors and as targets for therapy, presenting novel avenues for diagnosing and treating malignant skin tumors. Furthermore, the study highlights the need for further research to thoroughly comprehend the intricate interplay among different cathepsins and skin cancer subtypes, with the objective of refining targeted therapeutic approaches and enhancing patient outcomes.

## Acknowledgments

The authors thank all the patients in this research, all the scholars in this article, and all the teammates for supporting this research. This work was financially supported by a research project of the Science and Technology Development Project of Jilin Province, China (20200201315JC). The authors are also particularly grateful to their colleagues in The First Affiliated Hospital of Jilin University for their contributions. The authors thank Professor Zhiguang Yang, Director of the Department of Thoracic Surgery, the First Hospital of Jilin University, for the support of this study.

## Author contributions

**Conceptualization:** Fan Bu, Tianye Yang, Ji Lu.

**Formal analysis:** Kai Yu, Kang Chen, Li Rong.

**Data curation:** Changtao Ye, Kang Chen.

**Methodology:** Guixia Huang.

**Funding acquisition:** Ji Lu.

## Supplementary Material


